# The spleen in liver cirrhosis: revisiting an old enemy with novel targets

**DOI:** 10.1186/s12967-017-1214-8

**Published:** 2017-05-23

**Authors:** Liang Li, Mubing Duan, Weisan Chen, An Jiang, Xiaoming Li, Jun Yang, Zongfang Li

**Affiliations:** 1 National & Local Joint Engineering Research Center of Biodiagnosis and Biotherapy, The Second Affiliated Hospital, Xi’an Jiaotong University, No.157, Xiwu Road, Xi’an, 710004 Shaanxi China; 2 Liver and Spleen Diseases Research Center, Shaanxi Province, No.157, Xiwu Road, Xi’an, 710004 Shaanxi China; 3 Department of General Surgery, The Second Affiliated Hospital, Xi’an Jiaotong University, No.157, Xiwu Road, Xi’an, 710004 Shaanxi China; 4 Department of Pathology, The Second Affiliated Hospital, Xi’an Jiaotong University, No.157, Xiwu Road, Xi’an, 710004 Shaanxi China; 5 Department of Biochemistry and Genetics, La Trobe Institute for Molecular Science (LIMS), La Trobe University, Bundoora, VIC Australia

**Keywords:** Splenomegaly, Hypersplenism, Liver cirrhosis, Hepatic fibrogenesis, Hepatic immune microenvironment, Liver regeneration, Nanomedicine

## Abstract

The spleen is a secondary lymphoid organ which can influence the progression of multiple diseases, notably liver cirrhosis. In chronic liver diseases, splenomegaly and hypersplenism can manifest following the development of portal hypertension. These splenic abnormalities correlate with and have been postulated to facilitate the progression of liver fibrosis to cirrhosis, although precise mechanisms remain poorly understood. In this review, we summarize the literature to highlight the mechanistic contributions of splenomegaly and hypersplenism to the development of liver cirrhosis, focusing on three key aspects: hepatic fibrogenesis, hepatic immune microenvironment dysregulation and liver regeneration. We conclude with a discussion of the possible therapeutic strategies for modulating splenic abnormalities, including the novel potential usage of nanomedicine in non-surgically targetting splenic disorders for the treatment of liver cirrhosis.

## Background

The spleen is a secondary lymphoid organ containing specialized subsets of lymphocytes and myeloid cells, which are spatially organized within at least two functionally distinct regions. As the largest lymphoid organ in the body, the spleen contains highly elaborate tissue structures and is anatomically linked to the liver via the portal vein system [1, 2]. Clinically, liver cirrhosis is frequently accompanied by multiple complications including splenomegaly and hypersplenism [3, 4]. Previous studies have suggested that these splenic abnormalities may promote the progression of liver fibrosis to cirrhosis and exacerbate disease prognosis through multiple possible pathways [5–8]. Precise mechanisms, however, remain unclear and the overall impact of splenic contributions to the pathogenesis of liver cirrhosis remains to be clarified. Recently, additional mechanistic insights have been reported by several independent studies, prompting a timely review of the contributions of the spleen to liver cirrhosis progression.

In this review, we focus on the features and mechanisms of splenic dysregulation which may occur during liver cirrhosis. We first discuss the splenic alterations which potentially contribute to the cellular crosstalk between the spleen and liver. We propose that splenic contributions to liver cirrhosis mainly occur through the promotion of hepatic fibrogenesis, perturbation of the hepatic immune microenvironment and inhibition of liver regeneration. We further suggest that splenic immune cell alterations, especially in macrophages, monocytes and T cells, may be the most important perpetrator of this pathological process. Finally, we discuss the translational implications of these research findings, especially in application to nanomedicine and the discovery of novel and non-surgical strategies for the treatment of liver cirrhosis.

## The anatomy of the spleen

**Fig. 1:**
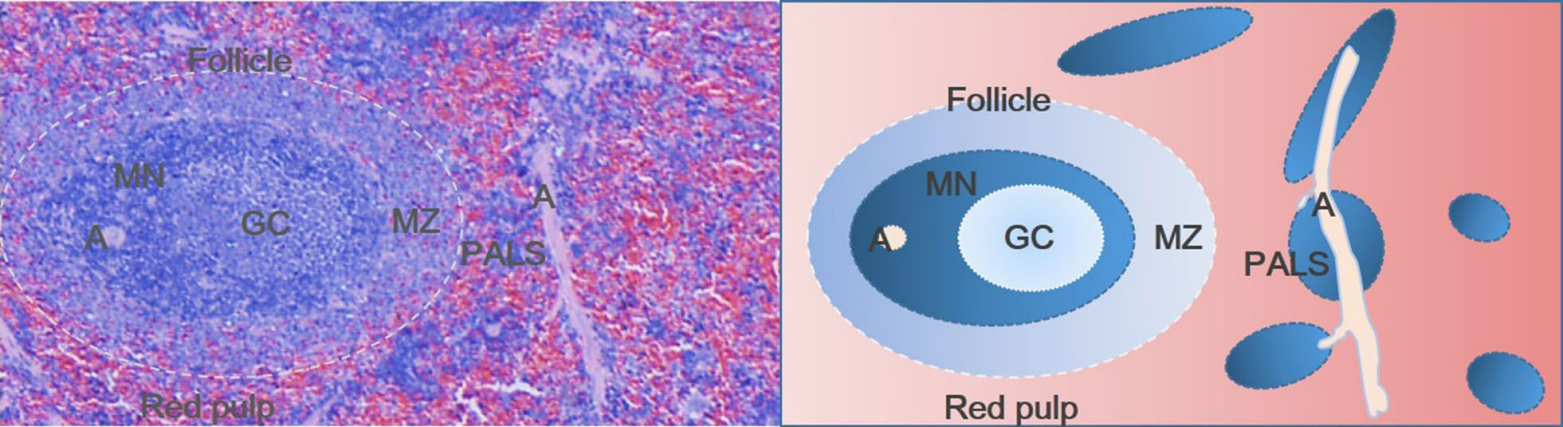
Diagram of the normal spleen parenchyma (rat). *Blue* regions depict features within the white pulp whilst *red* regions depict the red pulp. The white pulp is composed of multiple lymphoid follicles. A follicle consists of a reactive germinal center (GC) surrounded by a mantle zone (MN) and marginal zone (MZ). There are arterioles (A) adjacent to or within the white pulp. Lymphoid tissues envelope the arterioles to form periarteriolar lymphoid sheath (PALS). The white pulp regions are analogous to “buds” whereas the arterioles are analogous to “branches”. The red pulp is distributed as sinuses and solid-appearing cords, which separate the white pulp

The spleen is located within the left upper abdomen, adjacent to the stomach and beneath the diaphragm, with its appearance and size varying between different species [9]. The surface of spleen is encapsulated by visceral peritoneum and reflects inwards to form trabeculae that separate the splenic parenchyma from its supporting vasculature. The vessels of the spleen are organized as a ‘tree’ branching from the afferent splenic artery, with smaller arterioles converging into venous sinusoids within the red pulp, before collecting into the efferent splenic vein. Branch-like arterial vessels are sheathed by lymphoid tissues, which constitute the white pulp and consists of T cells (mainly in the periarteriolar lymphoid sheath, PALS) and B cells (mainly in the germinal center, GC). The marginal zone surrounds the white pulp and acts as an interface separating the white pulp from the red pulp [10]. Macrophages constitute the major immune cell population within the marginal zone and red pulp [11–13]. Stromal cells are also distributed within the marginal zone, and white and red pulp regions to support the localization of different immune cell subsets [14]. The compartments of the spleen are illustrated accordingly (Fig. 1) and more detailed reviews of the structure and functions of different splenic cell populations can be found elsewhere [10, 15]. As a structurally sophisticated and multi-functional lymphoid organ, the spleen exerts important effects on local and systemic immune responses, which have the potential to affect different tissues and organs.

## Splenic abnormalities in liver cirrhosis

During liver cirrhosis, splenomegaly and hypersplenism are relatively sub-fatal complications in the absence of bleeding varices. Splenic enlargement is one of the most palpable abnormalities accompanying liver cirrhosis, and frequently occurs in parallel with hypersplenism, which is thought to be a major cause of cytopenia and thrombocytopenia in cirrhotic patients [16]. Clinically, splenomegaly and hypersplenism are considered prominent though not progression-specific indications for liver cirrhosis given that they can manifest in other disease conditions [17]. The precise effectors of liver cirrhosis-associated splenomegaly and hypersplenism remain unclear, although altered hemodynamics, tissue injury and the release of inflammation-induced signaling molecules are now thought to play central roles.

### Splenomegaly

Spleen sizes can vary between cirrhotic patients by primary disease etiologies, with hepatitis C virus (HCV) infected and non-alcoholic hepatitis patients showing significantly larger organ dimensions compared to alcoholic hepatitis patients [18]. Histologically, chronic portal hypertension-induced splenomegaly features expanded white pulp and marginal zone areas and appears different to congestive splenomegaly, which is characterized by more prominent red pulp and less distinct white pulp regions [19, 20]. Clinically, splenomegaly has been associated with a poor prognosis in liver cirrhosis and utilized during radioactive or acoustic examinations as an index for the non-invasive assessment of esophageal varices and bleeding risks [1, 21, 22]. Splenic stiffness can also increase as splenomegaly advances [23]. Portal congestion is widely considered the initial cause of splenomegaly during liver cirrhosis [5, 24]. The subsequent changes in the enlarged spleen are complex and difficult to elucidate, considering the concurrent involvement of multiple cell populations in different compartments. Recently, Mejias et al. induced splenomegaly in rats using a partial portal vein ligation (PPVL) model of chronic portal hypertension. Interestingly, significantly increased activation of the mTOR signaling pathway was observed within the enlarged spleen. More importantly, mTOR inhibition using rapamycin profoundly ameliorated splenomegaly, causing a 44% decrease in spleen size [20]. Although the PPVL model more closely simulates human idiopathic portal hypertension (IPH), these findings remain suggestive for the study of cirrhosis-associated portal hypertension. In another study, Chen et al. utilized a rat model of portal hypertension induced by a combination of bile duct ligation (BDL) and PPVL. They reported that rapamycin-induced mTOR inhibition significantly decreased splenomegaly through the inhibition of lymphocyte proliferation, angiogenesis, fibrogenesis and tissue inflammation levels, which ultimately led to a decrease in portal pressure [25]. Consistent with Mejias et al., the findings from Chen et al. are insightful as the combination of BDL and PPVL models the augmentation of portal hypertension by biliary cirrhosis, which more closely mimics clinical cirrhosis conditions. Overall, the identification of portal hypertension-induced mTOR signaling alterations may be highly significant due to its central roles in immune cell modulation, angiogenesis and hepatic fibrogenesis [26–28]. Further investigations utilizing animal models of liver cirrhosis-associated portal hypertension will be required to confirm whether and how the mTOR signaling pathway may contribute to liver cirrhosis-associated splenomegaly.

### Hypersplenism

The incidence of hypersplenism has been reported to range from 11 to 55% in patients with cirrhosis and portal hypertension [29]. Hypersplenism often develops in parallel with splenomegaly. The mechanisms responsible for hypersplenism remain a matter of debate although recent studies iterate and corroborate that dysregulated immune cell responses may contribute to this process. In a study from Nomura et al., spleen samples from 26 patients with HCV-associated cirrhosis and hypersplenism who underwent splenectomy were studied by immunohistochemical staining and flow cytometry. They found that the splenic ratio of CD4^+^:CD8^+^ lymphocytes from these patients were higher compared to the control group (P = 0.06) whilst the ratio of FOXP3^+^:CD4^+^ was lower than the control group, implicating an increase in CD4^+^ T cell immune responses during hypersplenism [30]. A series of studies from our group have shown that splenic macrophages may also be hyperactivated and subsequently facilitate cirrhosis-associated hypersplenism [31–34]. We demonstrated that NF-kB p65/c-Rel signaling was significantly elevated in hypersplenic macrophages and promoted increased phagocytosis and secretion of both pro-inflammatory and pro-fibrogenic factors such as IL-1β, IFN-γ, TNF-α and TGF-β1 [35]. The source of this macrophage hyperactivation, however, remains unresolved. Interestingly, a recent study by Valdes-Ferrer et al. demonstrated that a major expansion of the inflammatory CD11b^+^Ly-6C^high^ monocyte pool occurs in the spleens of sepsis-induced mice which survive caecal ligation and puncture (CLP) and subsequently develop significant splenomegaly. Serum high-mobility group box 1 (HMGB1) levels were significantly elevated in surviving sepsis-induced mice for 4–6 weeks, with administration of recombinant HMGB1 to naive mice inducing similar splenomegaly, leukocytosis and splenocyte priming phenotypes as sepsis-induced survivors [36]. The administration of anti-HMGB1 monoclonal antibody also significantly attenuated splenomegaly and splenocyte priming levels in sepsis-induced survivors [36]. HMGB1 belongs to the family of damage-associated molecular patterns (DAMPs) and elevated serum and liver HMGB1 and other DAMPs levels have been widely reported in liver fibrosis and cirrhosis [37–39]. Consequently, it is possible for DAMPs such as HMGB1 to circulate and induce splenic immune cell dysregulation during these conditions.

Recent studies in the field of extracellular microvesicles have provided us with a new paradigm for understanding the crosstalk between the liver and spleen. In a study from Saunderson et al., splenic marginal zone macrophages were able to capture in vivo or injected B cell-derived exosomes through the binding of exosome surface α2,3-linked sialic acid to cell surface CD169 expressed on marginal zone macrophages. CD169^−/−^ mice showed an altered distribution of exosomes, with exosomes freely accessing the outer marginal zone rim of SIGN-R1^+^ macrophages and F4/80^+^ red pulp macrophages in contrast to wild type controls. Interestingly, CD169^−/−^ displayed enhanced CD8^+^ T cell responses to antigen-pulsed exosomes compared to wild type mice, suggesting that circulating exosomes may be selectively recognized and also captured by splenic macrophages for antigen presentation purposes [40]. Considering the extensive involvement of exosomes in the pathogenesis of liver diseases, especially liver fibrosis, a role for liver-derived exosomes in the development of hypersplenism should not be excluded [41–43]. Further study will be required to elucidate more detailed mechanisms of how the contributions of hepatic injury, inflammation and fibrogenesis may affect splenic homeostasis and contribute to hypersplenism.

## Splenic contributions to liver cirrhosis

An association between the liver and spleen has been proposed at least for three major reasons. Anatomically, both organs are important components of portal circulation. Histologically, the liver and spleen possess similar reticuloendothelial structures, which continuously participate in substance exchange and cellular migration [44]. Immunologically, both the liver and spleen play essential roles in immune homeostasis as well as pathogen clearance. Thus, the concept of a liver-spleen axis has been proposed as an intersection linking immunity, pathogen clearance and metabolism in various conditions including chronic liver diseases [45]. Previous studies have unanimously implicated innate and adaptive immune cells in development of liver fibrosis or cirrhosis [46–49]. However, direct evidence for the involvement of splenic immune cells or spleen-derived factors has only recently emerged, suggesting that splenic contributions to hepatic fibrogenesis, hepatic immune microenvironment dysregulation and the disruption of liver regeneration may be responsible.

### Splenic contributions to hepatic fibrogenesis

During liver fibrosis, hepatic stellate cells (HSCs) and Kupffer cells (KCs) act as the initial effectors of collagen deposition and inflammation modulation with the aid of the pro-fibrogenic cytokine transforming growth factor beta 1 (TGF-β1) [50, 51]. Previous studies have reported splenic TGF-β1 production in the context of liver cirrhosis and hypersplenism and emphasized its critical role in the development of hepatic fibrogenesis. Splenic macrophages have been suggested as one source of TGF-β1. In a study by Akahoshi et al., a rat model of liver cirrhosis was induced by intraperitoneal injection of thioacetamide (TAA) for 24 weeks, and followed by either a splenectomy or sham operation. Splenic red pulp macrophages were suggested as a major source of TGF-β1 in this study, and splenectomy was reported to decrease serum TGF-β1 levels significantly whilst improve liver fibrosis and regeneration parameters [6]. A recent study by Asanoma et al. detected increased TGF-β1 expression from the resected spleens of liver cirrhotic patients by immunofluorescence staining, and reported it to be significantly correlated with the progression of liver cirrhosis. TGF-β1 immunofluorescence further overlapped with the staining of CD68, a pan marker of tissue macrophages [52]. Overall, the studies from Akahoshi et al. and Asanoma et al. both implicate that splenic involvement during hepatic fibrogenesis may be mediated through splenic macrophage production and secretion of TGF-β1. Thus, further studies elucidating the explicit mechanisms driving splenic macrophage TGF-β1 production during liver cirrhosis in the presence or absence of hypersplenism will be of great interest.

### Splenic influences on the hepatic immune microenvironment

As the largest lymphoid organ in the body, the spleen contains multiple immune cell subsets which are differentially distributed within white and red pulp and marginal zone regions. B cells are mainly distributed within splenic follicles whilst T cells predominate in the white pulp regions. By contrast, DCs and macrophages are located predominately in the marginal zone (MZ) and red pulp regions. Immune cell migration between these different regions is necessary for splenic maintenance of immune cell homeostasis, tolerance and pathogen clearance [13, 53–55]. Interestingly, the red pulp has been revealed to maintain a reservoir of monocytes capable of rapidly migrating into injured tissues and mediating local inflammatory responses [56]. Similarly, T lymphocytes and some innate lymphoid cell (ILC) subsets have also been reported to be capable of splenic extravasation [57, 58].

**Fig. 2:**
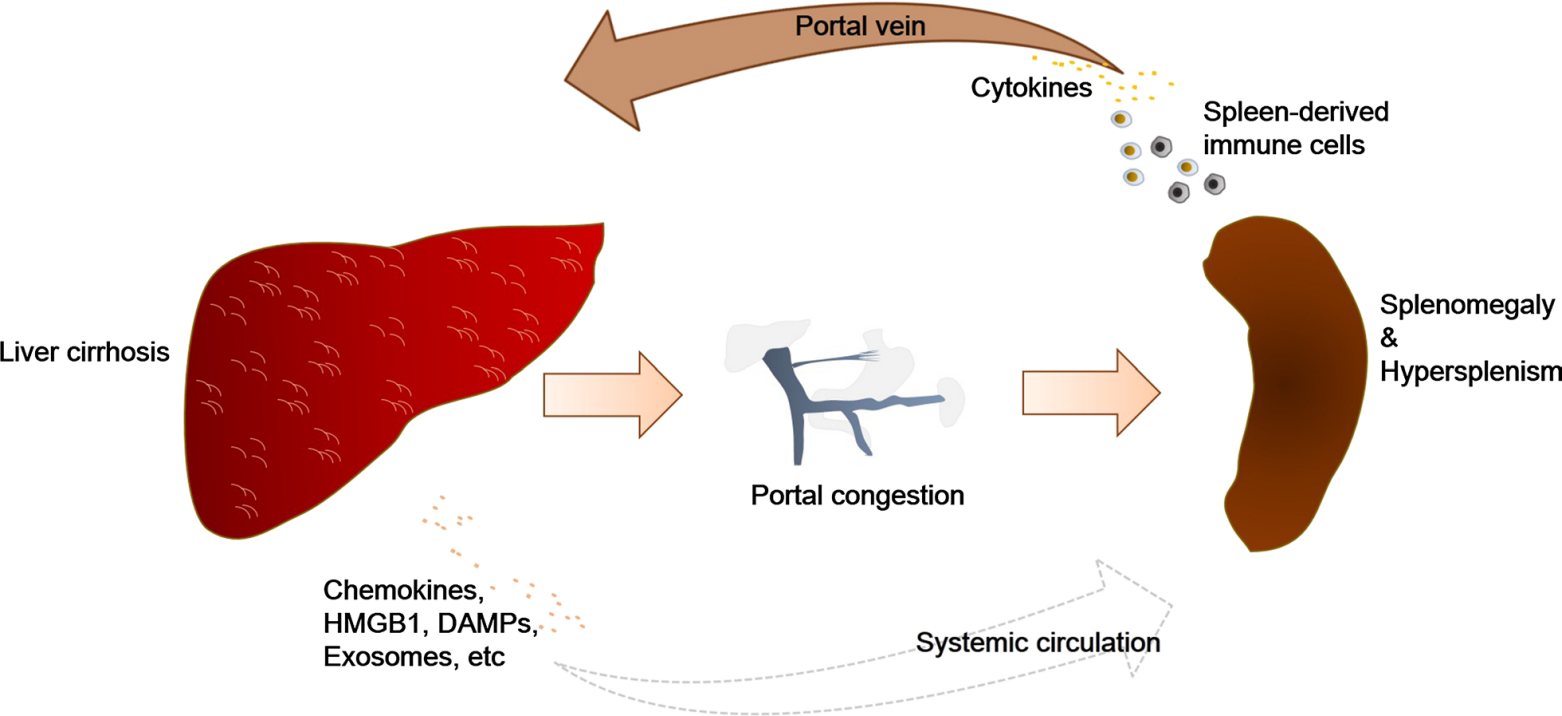
Diagram of the liver and spleen crosstalk pathways during liver cirrhosis. During liver cirrhosis progression, spleen-derived immune cells and cytokines may travel into the injured liver via portal blood flow. At the same time, liver cirrhosis also contributes to portal hypertension, which leads to the congestion of the portal system and may give rise to splenomegaly and hypersplenism. The cirrhotic liver may also release chemokines, DAMPs like HMGB1, or exosomes into the circulation, which trigger the activation and/or migration of splenocytes

As the liver and spleen are closely associated via the portal vein system, it is more likely for the spleen to exert its influences on the hepatic immune microenvironment by cell migration or the secretion of splenic soluble factors via portal vein blood flow (Fig. 2). In a recent study by Yada et al., splenectomy significantly increased the hepatic accumulation of Ly-6C^low^ monocytes or macrophages in a thioacetamide-induced murine model of liver cirrhosis with hypersplenism, implicating a role for the splenic control of hepatic monocyte or macrophage phenotypes [59]. Although no splenic monocyte tracing studies have been conducted in the setting of liver fibrosis or cirrhosis, the findings from Yada et al. are potentially suggestive when considering the directional movement of red pulp monocyte reservoirs towards cardiac lesions [56].

Studies focusing on T cells have also shed light in another direction. In a study of *Schistosoma japonicum* associated liver fibrosis by Romano et al., splenomegaly was found to be correlated with higher levels of FOXP3^+^ regulatory T cells (Tregs) in the blood and increased liver fibrosis severity. Splenectomy decreased Treg cell and hepatic fibrogenesis levels, implicating a role for the splenic modulation of the liver via alterations in T cell subsets [60]. More interestingly, in a whole genome microarray analysis conducted by Burke et al. [61] following *Schistosoma japonicum* infection, lymphocyte and monocyte chemokines and cell adhesion molecules were significantly upregulated in the liver whilst concurrently downregulated or unaltered in the spleen, possibly suggesting a recruitment of effector cells from the spleen to the liver. Another study by Tanabe et al. has explicitly pointed that splenic T cells may migrate into the fibrotic liver to promote hepatic fibrogenesis. They reported a decrease in splenic CD4^+^ T cell numbers following carbon tetrachloride (CCl4) or thioacetamide (TAA) induced liver injury in BALB/c mice. Splenectomy shifted the liver helper T cell (Th) Th1/Th2 balance towards Th1 and suppressed the progression of liver fibrogenesis. Adoptive transfer of GFP^+^ splenocytes into the spleens of syngeneic wild type mice following fibrosis induction led to the accumulation of GFP^+^CD4^+^ lymphocytes with a Th2 phenotype in the diseased liver. The transfer of GFP^+^ splenocytes into the portal vein of syngeneic splenectomized mice abolished the suppressive effects of splenectomy on liver fibrosis, highlighting that splenic contributions to liver fibrosis may be directly mediated by the migration of splenic immune cells into a subsequently altered hepatic immune microenvironment [57]. Sophisticated in vivo tracing studies will be needed, however, to confirm the impact of splenic immune cell migration and/or secreted soluble factors on the hepatic immune microenvironment during liver fibrosis and cirrhosis.

### Splenic modulations of liver regeneration

Although the liver normally possesses a huge regenerative capacity, this capacity is compromised during liver injury or resection, especially in the presence of severe chronic liver injury with marked fibrosis and tissue architecture aberrations [62, 63]. Liver regeneration is frequently overwhelmed by fibrogenesis in chronic liver diseases [64]. During chronic liver injury, hepatic progenitor cells (HPCs) act as the regenerative source for hepatocyte or cholangiocyte replenishment [65, 66]. Liver resident macrophages, also known as KCs, have also been shown to promote the differentiation of progenitor cells through debris engulfment-induced Wnt3a signaling in chronic liver diseases [67, 68]. Recently, residential KCs and recruited monocyte-derived macrophages have been both found to play central roles in liver regeneration, most likely through a mechanism involving KC–monocyte crosstalk and the consequent activation of HPCs [69]. No study has yet to determine, however, whether splenic monocyte-derived macrophages can directly influence KCs or HPCs to alter liver regeneration capacities during chronic liver injury.

Several studies point towards the potential of splenic contributors to impact liver regeneration capacities. Studies from Murata et al. and Yamada et al. have demonstrated that splenectomy improved liver regeneration in cirrhotic animals and patients respectively [70, 71]. In a study by Ueda et al. of rats undergoing major hepatectomy with or without splenectomy, early stage splenic red pulp TGF-β1 production and secretion into the portal blood was suggested to exert an inhibitory effect on liver regeneration. Splenectomy reversed this inhibition and enhanced the regeneration of hepatocytes [72]. In a separate study by Lee et al., which used rats undergoing a 70% hepatectomy with or without splenectomy, decreased TGF-β1 and increased hepatocyte growth factor (HGF) levels in the portal vein occurred concurrently to increase liver regeneration following splenectomy. Liver TGF-β1 and HGF receptor levels were also correspondingly regulated by splenectomy in this study [73]. Altogether, these studies suggest a role for the spleen in the inhibition of liver regeneration via TGF-β1 upregulation and HGF downregulation and its subsequent disruption of the hepatic pro-versus anti-regenerative signaling balance. However, more detailed mechanistic pathways remain to be resolved.

Recent studies using stem cell therapy in chronic liver diseases have also provided some clues regarding the role of the spleen in hepatic regeneration. In a study by Iwamoto et al., splenectomy enhanced the repopulation of adoptively transferred bone marrow cells in cirrhotic liver and decreased collagen deposition through the upregulation of MMP9 expression in transferred bone marrow cells [74]. In a separate study of liver cirrhosis in rats, Tang et al. reported that splenectomy improved the efficiency of adipose tissue-derived mesenchymal stem cell transplantation into the liver by enhancing liver SCF-1 (stromal cell-derived factor-1) and HGF expressions [75]. Further studies, however, will be required to characterize whether and how spleen-derived cells or soluble factors can mediate direct effects on liver-recipient stem cell expansion or hepatic progenitor cell behavior.

## Targeting spleen for the treatment of liver cirrhosis

Targeting splenic abnormalities may be crucial for the management and treatment of liver cirrhosis due to their multiple pathophysiological associations with cirrhotic disease progression. In the past decades, splenectomy has been utilized to ameliorate the fatal complications of cirrhosis-associated portal hypertension. An alternative to splenectomy, however, is required for many patients with precluding conditions such as thrombocytopenia. Although such alternatives remain lacking, advances in the field of nanomedicine, especially the usage of nanoparticles for drug delivery and cell targeting, may provide us with novel avenues for targeted therapies.

### Splenectomy

Splenectomy has been traditionally performed to treat liver cirrhosis for the alleviation of portal hypertension. The utility of splenectomy is well accepted and may be especially important for the treatment of fatal complications such as bleeding esophageal or gastric varices. Apart from its effects on portal hypertension and hypersplenism, splenectomy has also been reported by many groups to be an efficient method for improving liver function and the prognosis of esophageal varices [8, 76]. Previous studies have also reported splenectomy to increase the efficacy of liver transplantation and improve the prognosis of hepatocellular carcinoma [77–79]. A study by Ogawa et al. has also suggested splenectomy as a supplemental treatment for anti-HCV therapy in combination with interferons and other pharmaceuticals, which is consistent with our own finding [80, 81]. Although animal studies suggest that splenectomy can ameliorate collagen deposition, improve liver function and regeneration capacity and enhance the therapeutic efficacy of stem cell transplantations [6, 59, 75], many cirrhosis patients present with contraindications which preclude splenectomy. Thus, it remains critical for us to identify alternative novel and non-surgical methods which can target the spleen for the treatment of liver cirrhosis.

### Non-surgical therapies targeting the spleen

Previous studies suggest that there is potential therapeutic value in targeting splenic mTOR signaling for the amelioration of splenomegaly and hypersplenism. Inhibition of splenic macrophage TGF-β1 production may be another therapeutic target in the treatment of liver cirrhosis progression. Therapies selectively modulating splenic macrophage or T cell activation may also be useful as these immune cell aberrations have been linked to hepatic immune microenvironment dysregulation and the enhancement of hepatic fibrogenesis. One of the biggest obstacles to these approaches has traditionally been the absence of spleen-selective delivery options for therapeutic agents. This problem, however, may be circumvented by the use of nanoparticles for novel and targeted drug delivery. In a study of nanoparticle distribution during LPS-induced systemic inflammation in mice, the spleen showed increased retention of carboxylated polystyrene latex bead nanoparticles of different sizes compared to all other organs studied. Nanoparticle retention localized mostly to the splenic marginal zone and red pulp to a lesser extent, with most nanoparticles found to be ingested by splenic macrophages. The uptake of nanoparticles by splenic macrophage was size dependent, with 20–100 nm particles preferentially engulfed by CD68^+^ marginal zone macrophages, 100–500 nm particles engulfed by CD169^+^ metallophilic marginal zone macrophages and 500 nm particles engulfed by F4/80^+^ red pulp macrophages. This size dependence may be caused by the increased permeability of splenic vessels during inflammatory conditions, and highlights one interesting prospect for the selective targeting of differential splenic regions [82].

An excellent review of the principles of nanoparticle design can be found elsewhere [83]. In a recent study by Hu et al., poly(lactic-co-glycolic acid) nanoparticles were fashioned with membranes (containing both lipid and protein components) isolated from red blood cells in order to prolong nanoparticle retention time in the circulation. This approach may also be applicable for spleen selective drug targeting as splenic macrophages actively phagocytose red blood cells during hypersplenism and liver cirrhosis [84]. Research into the targeting of splenic and liver Leishmaniasis infections using nanoparticles may also be of potential strategic use [85, 86]. Standardized studies in animal models and pre-clinical trials measuring the efficacies of nanoparticle-based therapeutic agents will also be required for the development of novel spleen-selective therapeutic agents. In addition to nanoparticles, recombinant exosomes may also be useful for splenic targeting given the potentially selective capture of exosomes by splenic macrophages [40, 87, 88].

In addition to nanoparticle-based therapeutic agents, the crosstalk between the spleen and nervous system should also be considered in order to develop a comprehensive spleen-modulating approach for treating liver cirrhosis. A dense network of sympathetic noradrenergic fibers are closely associated with the splenic artery and branch into the white pulp and consequently T and B cell regions. Secreted neuropeptides may also directly affect the behaviour of splenic immune cells, and this may also need to be factored when designing spleen modulating therapies [89, 90]. Furthermore, the inhibition of liver injury-induced signaling molecules that perturb spleen homeostasis, such as HMGB1 or other DAMPs, may also be beneficial.

## Conclusions

**Fig. 3:**
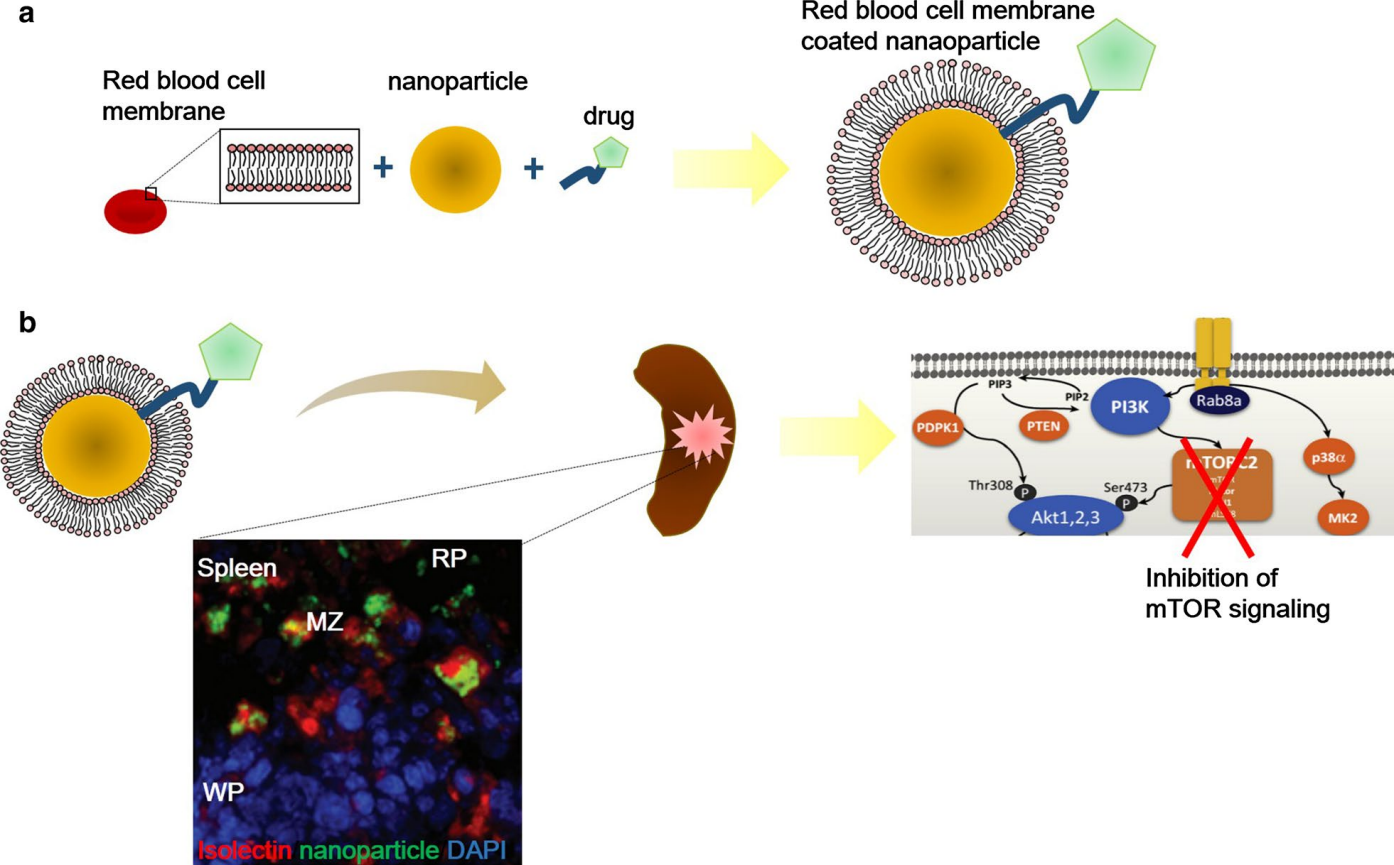
Diagram of the potential usage of nanoparticles for splenic targeting. Coating nanoparticles with red blood cell-like membrane may be helpful for increasing nanoparticle uptake by splenic leukocytes. These nanoparticles should then be conjugated with drugs targeting specific signaling pathway activated in spleen. **a** Drug-conjugated nanoparticles may then be ingested by macrophages within the red pulp or marginal zone upon circulation and distribution into the spleen. **b** These drugs then inhibit pathologically activated signals, such as mTOR signaling, to reverse liver-injury induced splenic perturbations. *WP* white pulp, *MZ* marginal zone, *RP* red pulp. The immunofluorescence image is from an article by Chen K-H and the schematic diagram of mTOR signaling is from a review paper by Sukhbaatar et al. [82, 91]

Numerous recent findings have greatly amended our understanding of the splenic contributions to liver cirrhosis. In particular, the discovery that the spleen can act as an inflammatory monocyte reservoir provides a new paradigm for understanding the immune-mediated links between the spleen and other organs. Recent studies on the development of splenomegaly and hypersplenism and the roles of spleen-derived cells or soluble factors in liver cirrhosis progression have greatly increased our comprehension of the crosstalk between the liver and spleen. Splenic characterization in the context of liver cirrhosis will be important for the future management and treatment of liver cirrhosis. New developments in the field of nanomedicine may provide us with improved strategies for targeting the spleen, given its vast reticuloendothelial system and abundant phagocyte distributions (Fig. 3). Future studies, however, will be required to characterize the precise mechanistic pathways which mediate spleen and liver crosstalk during disease progression from liver fibrosis to cirrhosis. This may potentially enable the development of new therapeutic strategies for splenic modulation during the management and treatment of liver cirrhosis, especially in circumstances where the option of splenectomy is precluded. Overall, a comprehensive understanding of the molecular and cellular pathways controlling splenic homeostasis and pathophysiology will be critical for the development of new therapies against liver cirrhosis.

## Key points:

1. Dysregulated mTOR signaling following portal hypertension is associated with the development of splenomegaly and hypersplenism.
2. Splenic immune cell dysregulation during liver cirrhosis may be induced by liver-derived HMGB1, DAMPs or exosomes.
3. Splenic contributions to liver cirrhosis progression may occur via the modulation of hepatic fibrogenesis, immune microenvironment dysregulation and liver regeneration, through chemotactic egress of spleen-derived cells or the release of splenic soluble factors.
4. Although splenectomy alleviate liver cirrhosis, the use of non-surgical methods like nanomedicine may be more valuable for the future treatment of liver cirrhosis.

## Abbreviations

mTOR: mammalian target of rapamycin
FOXP3: forkhead box P3
NF-κB: nuclear factor kappa-light-chain-enhancer of activated B cells
HMGB1: high-mobility group box 1
DAMPs: damage associated molecular patterns
TGF-β1: transforming growth factor β1
HSC: hepatic stellate cell
KC: Kupffer cell
HGF: hepatocyte growth factor
